# Supplementary material for: The interactome of intact mitochondria by cross-linking mass spectrometry provides evidence for coexisting respiratory supercomplexes
Source: Mol Cell Proteomics. 2017 Dec 8;17(2):216–32. doi: 10.1074/mcp.RA117.000470 (PMC5795388; doi:10.1074/mcp.RA117.000470)
Supplement: Supplemental Data [file supp_RA117.000470_133922_0_supp_23978_fzffwf.zip › spectra_annotation/mito_spectra_annotation/308-1-7-1-56-1.pdf]

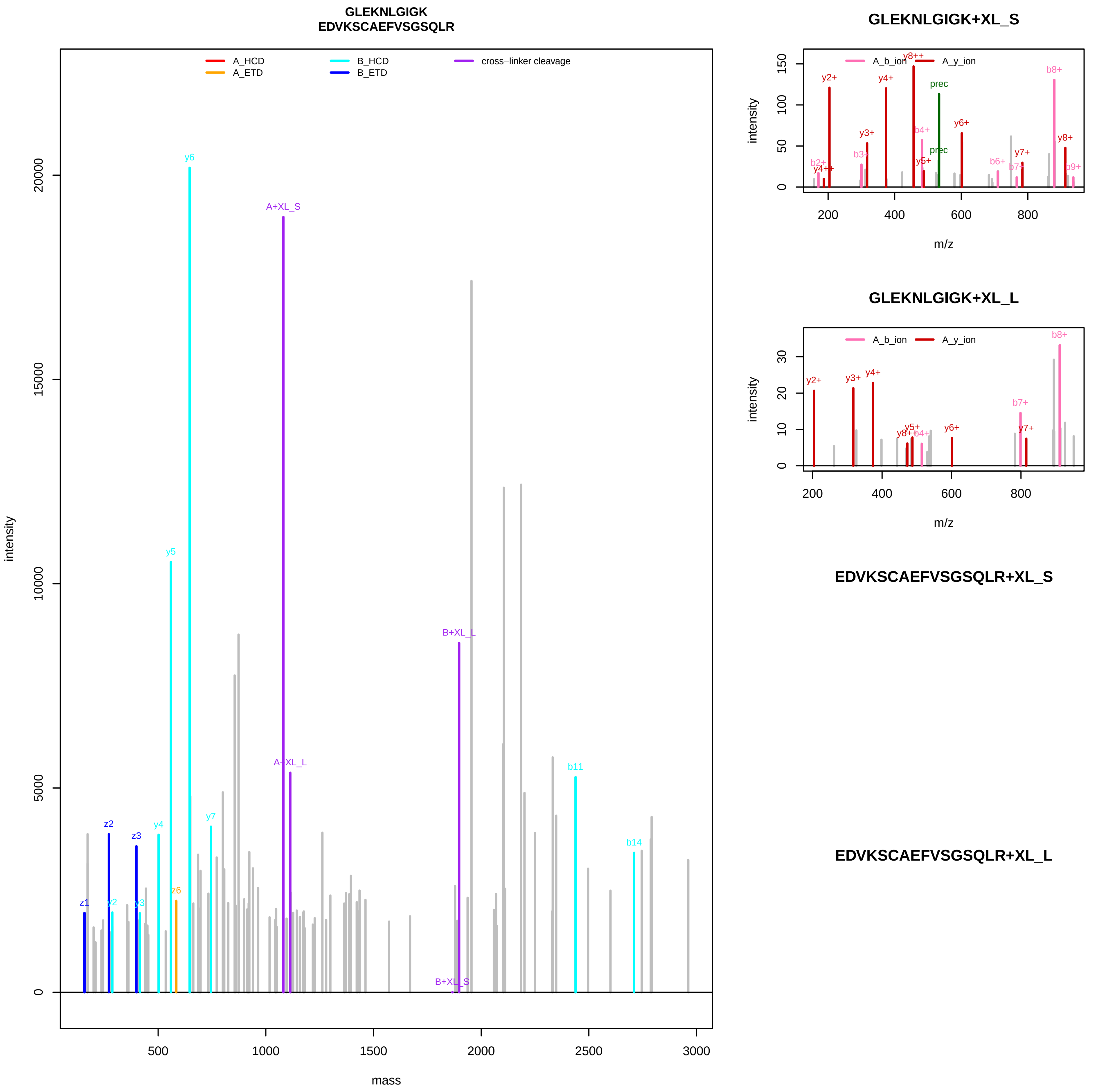
GLEKNLGIGK
EDVKSCAEFVSGSQLR
A_HCD
A_ETD
B_HCD
B_ETD
cross−linker cleavage
y6
A+XL_S
y5
B+XL_L
A+XL_L
b11
b14
z1
z2
y2
z3
y3
y4
z6
y7
B+XL_S
intensity
mass
GLEKNLGIGK+XL_S
A_b_ion
A_y_ion
y2+
y4+
y8++
prec
y3+
b4+
y6+
b2+
y4++
b3+
y5+
b6+
b7+
y7+
b8+
y8+
b9+
m/z
GLEKNLGIGK+XL_L
y5+
y8++
EDVKSCAEFVSGSQLR+XL_S
EDVKSCAEFVSGSQLR+XL_L
